# *LILRA3* Is Associated with Benign Prostatic Hyperplasia Risk in a Chinese Population

**DOI:** 10.3390/ijms14058832

**Published:** 2013-04-24

**Authors:** Yang Jiao, Li Wang, Xin Gu, Sha Tao, Lu Tian, Rong Na, Zhuo Chen, Jian Kang, Siqun L. Zheng, Jianfeng Xu, Jielin Sun, Jun Qi

**Affiliations:** 1 Department of Urology, Xinhua Hospital, Medical School of Shanghai Jiaotong University, Shanghai 200092, China; E-Mails: suntribe@yahoo.cn (Y.J.); guxin111@163.com (X.G.); kjjjjd@yahoo.com (J.K.); jasonqi@sh163.net (J.Q.); 2 Center for Cancer Genomics, Wake Forest University School of Medicine, Winston-Salem, NC 27157, USA; E-Mails: liwan@wakehealth.edu (L.W.); shtao@wakehealth.edu (S.T.); lu.tian.mes@gmail.com (L.T.); narong.hs@gmail.com (R.N.); zhchen@wakehealth.edu (Z.C.); szheng@wakehealth.edu (S.L.Z.); jisun@wakehealth.edu (J.S.); 3 Center for Genomics and Personalized Medicine Research, Wake Forest University School of Medicine, Winston-Salem, NC 27157, USA; 4 Fudan-VARI Center for Genetic Epidemiology, Fudan University, Shanghai 200422, China; E-Mail: jxu@wakehealth.edu

**Keywords:** benign prostatic hyperplasia (BPH), inflammation, *LILRA3*, single nucleotide polymorphism (SNP), Chinese

## Abstract

A recent prostate cancer (PCa) genome-wide association study (GWAS) identified rs103294, a single nucleotide polymorphism (SNP) located on *LILRA3*, a key component in the regulation of inflammatory inhibition, to be significantly associated with PCa risk in a Chinese population. Because inflammation may be a common etiological risk factor between PCa and benign prostatic hyperplasia (BPH), the current study was conducted to investigate the association of rs103294 with BPH risk. rs103294 was genotyped in a Chinese population of 426 BPH cases and 1,008 controls from Xinhua Hospital in Shanghai, China. Association between rs103294, BPH risk and clinicopathological traits were tested with adjustment for age. rs103294 was significantly associated with BPH risk with a *p*-value of 0.0067. Individuals with risk allele “C” had increased risk for BPH (OR = 1.34, 95% CI: 1.09–1.66). Stratified analysis revealed a stronger association risk for younger patients who are below 72 years old (OR = 1.51, 95% CI: 1.06–2.16). Our study represents the first effort to demonstrate that *LILRA3* gene is significantly associated with BPH risk in a Chinese population. Our results support a common role of inflammation in the development of PCa and BPH. Additional studies are needed to further evaluate our results.

## 1. Introduction

Benign prostatic hyperplasia (BPH) is a common disease that is increasingly prevalent in men over the age of fifty. It is the fourth most common diagnosis in older men [1]. A quarter of the men in their 50s are affected, a third in their 60s and half of the men past their 80s [2]. BPH is a nonmalignant enlargement of the prostate gland, clinically manifesting as lower urinary tract symptoms (LUTS) or acute urinary retention (AUR). Current management options for BPH include medications, minimally invasive therapies, and prostate surgery with continued surveillance [1,3].

The causes of BPH are not fully known, but the overgrowth of smooth muscle tissue and glandular epithelial tissue is attributed to various factors such as aging, late activation of cell growth, hormones, and genetic factors. Diagnostic factors include age, prostate size, weight, prostate-specific antigen level, and severity of symptoms [3]. Previous studies have observed important anatomic, pathologic, and genetic links, in addition to well-established epidemiological associations, between prostate cancer (PCa) and BPH [4]. Although PCa and BPH form in different areas of the prostate and present in two distinct pathogenetic pathways, studies have suggested several common characteristics between PCa and BPH, including incidence and prevalence rise with increased age, both conditions are hormone dependent and both diseases are associated with prostatic inflammation [4–7].

Prostatic inflammation contributes to the development and progression of BPH, rather than occurring in response to altered tissue architecture, suggesting an immune inflammatory nature [2]. Single-nucleotide polymorphisms (SNPs) associated with inflammation have been implicated in the development of PCa [8,9]. However, very few studies had been conducted to test for association between genes involved in inflammation and BPH risk [8,10,11]. A recent genome-wide association study (GWAS) in PCa identified rs103294 to be significantly associated with PCa risk at a genome-wide significant level (*p* = 5.34 × 10^−16^) in the Chinese population [9]. rs103294 is located on *LILRA3* gene, which is a key regulator of the inflammatory response. Due to the shared role of inflammation in both PCa and BPH, we conducted the current study to evaluate the association of the *LILRA3* SNP and BPH risk. Our study represents the first study to evaluate the role of *LILRA3* in BPH development and progression in a Chinese population.

## 2. Results and Discussion

### 2.1. Demographic and Clinical Information

Demographic and clinical information for 426 cases and 1008 controls with genotypes are presented in Table 1. Aggressive BPH cases comprised (43.0%) and nonaggressive cases (57.0%). Mean age was higher for cases (71.9 ± 7.9 years) compared to controls (61.2 ± 9.0 years) and was adjusted in the association tests. Baseline clinicopathological parameters are also presented in Table 1.

### 2.2. Genetic Association Results

Genotype distributions for the SNP were in Hardy Weinberg Equilibrium (HWE) in both case and control groups (*p* > 0.05, data not shown). rs103294 had missing rates smaller than 5% (data not shown). SNP association results are shown in Table 2. rs103294 showed a significant association with BPH (*p* = 0.0067). Risk allele “C” of rs103294 was associated with a 1.34 fold increased risk of BPH (95% CI: 1.09–1.66). It was not associated with aggressive BPH (*p* = 0.28).

Association results with clinicopathological traits are presented in Table 3. rs103294 showed no significant association with the clinicopathological traits(All *p* > 0.05). Stratified analyses (Table 4) showed a stronger effect for rs103294 for patients under 72 years of age (OR = 1.51). Subjects over the age of 72 showed a weaker effect (OR = 1.27). Similar effects were observed for patients with different total prostate volume (TPV) and International Prostate Symptom Score (IPSS) values (Table 4).

### 2.3. Discussion

In this study, we investigated the association of rs103294, a recently identified PCa risk-associated SNP through GWAS study in Chinese, with BPH risk in a Chinese population of 426 cases of BPH and 1,008 controls. In our study, rs103294 was significantly associated with BPH risk (*p* = 0.0067). Our study is among the first efforts which demonstrate a critical role of *LILRA3* gene in BPH development.

Currently, the role of gene polymorphisms in the development of BPH remain unclear inconsistent due to BPH’s polygenic and multifactorial nature [11]. Various reasons such as the high prevalence of the disease and demographic trends towards advanced age indicate that genetic markers for clinically determining BPH are relevant and needed [12]. Importantly, SNPs may regulate and predispose disease initiation or progression of chronic prostatic diseases. Though candidate gene and genetic linkage approaches have yielded various candidate genes for BPH, such as *CYP3A4* for steroid-metabolism pathways, the androgen receptor (*AR*) gene and the *SRD5A* steroid reductase genes, they have been unsuccessful in restricting potential candidates due to inconsistent results [11]. In addition, no GWAS studies have been conducted for BPH related phenotypes. Therefore, identifying genetic factors that are associated with BPH phenotypes are very important in explaining the genetic component of this common disease.

Due to the potential link between PCa and BPH, our study evaluated a SNP proven to have significance with PCa risk at a genome-wide association level with potential functional implication for BPH. In a recent Chinese PCa GWAS study, rs103294 was found to be significantly associated with PCa risk (*p* = 5.34 × 10^−16^) in a combined study population of 4484 PCa cases and 8,934 controls [9]. Risk allele “C” of rs103294 was associated with 1.28 fold of increased risk of prostate cancer [9]. In our study, the risk allele “C” of rs103294 was also associated with increased risk of BPH. rs103294 is located in the leukocyte immunoglobulin-like receptor (LIR) gene cluster at 19q13.4, between the region upstream of *LILRA3* and downstream of *LILRB2*. rs103294 is in strong linkage disequilibrium (LD) (r^2^ = 0.83) with a germ-line deletion of six of seven exons of the functional domains of *LILRA3*, an inflammatory regulatory gene. *LILRA3* is the only secretory leukocyte immunoglobulin-like receptor (*LILR*) in the LIR cluster, which may regulate the inhibitory immune response induced by *LILRB1*, *LILRB2*, and other molecules like *LILRA1* [13]. Thus, *LILRA3* is likely important for regulating the inflammatory response.

*LILRA3*’s regulation of the inflammatory response is important because epidemiological data shows an overlap between PCa and BPH through inflammation. Emerging evidence shows that inflammation can promote chronic prostatic diseases by inducing carcinogenesis by causing cell and genome damage. Inflammation has been highly suggestive for prostate growth in both BPH or PCa [5,14,15] and has also been implicated in the progression of BPH [15,16], although the etiology and epidemiology of BPH is complex and not fully understood. A previous study of the role of interleukin 10 (*IL10*), a multifunctional cytokine with anti-inflammatory and anti-angiogenic properties, in BPH showed that *IL10* SNPs play an important role in the process of prostate inflammation and are associated with clinicopathological traits such as TPV and PSA. However, the study did not evaluate BPH susceptibility risk because association studies were conducted within cases only [10]. Therefore, our study is one of the first to show the risk contribution of inflammation in the development of BPH. In addition, because of the important role of rs103294 in PCa and the potential link between PCa and BPH, the association observed in our population more likely represents a true association.

Although rs103294 is significantly associated with BPH risk, it was not associated with severity of BPH in our study population. In addition, association results for clinicopathological traits did not reveal potential link with rs103294, further indicating that it may only contributes to BPH susceptibility. Additional studies are needed to identify such markers for severity in order to provide improved risk prognosis for BPH. Clarification risk of progression will lead to more differentiated diagnosis of older men with BPH [3]. In addition, we further examined potential stratified effects by age, TPV and IPSS in order to test for significant effects in subgroups such as younger and older age groups. Subjects under the age of 72 were found to have more of a predisposition (OR = 1.51) towards the risk effect of rs103294 than subjects over the age of 72 (OR = 1.27). The significant association of a genetic effect amongst a younger population supports the role of genetics in determining the etiology of BPH.

A potential limitation to our study is that only one SNP for *LILRA3* gene was chosen to be evaluated. However, through a fine-mapping and imputation effort, rs103924 was the leading SNP in this genomic region [9]. No other SNPs remained significant if adjusting for rs103294 in the statistical model, suggesting that no additional independent prostate cancer risk-associated loci existed at this region [9]. Thus, rs103294 is able to capture the majority of the genetic information in the LIL gene cluster at 19q13.4.

## 3. Experimental Section

### 3.1. Study Subjects

All subjects were of a Chinese Han ancestry. A total of 426 BPH cases were enrolled from the department of Urology, Xinhua Hospital (Shanghai Jiao Tong University School of Medicine), Shanghai, China, during the period of July 2010 to July 2012. Patients were included in the study with informed consent prior to qualifying study inclusion criteria.

The population underwent the following investigations: International Prostate Symptom Score (IPSS), including the quality of life question (IPSS-Q1); postvoid residual volume (PVR) measurement by transabdominal ultrasonography, determination of prostate size by transrectal ultrasonography; a serum prostate-specific antigen (PSA) determination; liver and renal function; blood glucose level; and routine urine examination. Inclusion criteria for BPH patients at baseline were benign prostatic enlargement (BPE) with LUTS of age > 45 years, prostate size > 30cm, IPSS > 7 and PVR volume ≤ 1500 mL. Patients with PSA < 4 ng/mL were included in the study and some patients with PSA ≥ 4 were included only after DRE (digital rectal examination), true-cut biopsy for confirmation for lack of PCa, and long-time follow-up visit of stabilized PSA. Exclusion criteria were history of urinary tract infection (UTI), previous lower tract surgery or procedures and neurogenic bladder dysfunction.

All eligible subjects were treated with combined therapy of 4 mg α-adrenergic blockers (doxazosin) and 5 mg of 5α-reductase inhibitors (finasteride) once daily. The length of treatment exceeded at least nine months. After adequate treatment time, if subjects suffered from a significant increase in the IPSS score; continuous decrease in maximum urinary flow rate or BPH related complications (AUR; bladder stone or recurrent hematuria, *etc.*) and had to receive operation by surgery, they were defined as “aggressive BPH” patients. In contrast, patients without complaints of aggravated symptoms, as well as no indications of operation by surgery were defined as a non-aggressive group. Thus, 184 aggressive and 242 non-aggressive BPH cases were defined. The detailed information for controls used was previously reported in Ma *et al.* [17]. Briefly, 1008 community males were used as controls in the current study. All the controls were collected from April 2010 to November 2010 in Shanghai, China.

### 3.2. SNP Selection

rs103294 was recently reported to be associated with PCa risk at a genome-wide significant level in a Chinese population [9] and was evaluated in the current study.

### 3.3. Genotyping

The SNP was genotyped for all study subjects using the MassARRAY iPLEX system (Sequenom, Inc., San Diego, CA, USA) at Fudan University in Shanghai, China. Two duplicates and two water samples were included in each 96-well plate as PCR-negative controls. All assays were performed in a blinded fashion. Genotyping missing rates was 0.7%.

### 3.4. Statistical Analysis

The genotype distributions for the SNP were tested for Hardy-Weinberg equilibrium (HWE). The main effects of the SNP for BPH risk were estimated using a logistic regression model, assuming an additive mode of inheritance, adjusting for age. Quantitative clinicopathological traits including IPSS, TPV, total prostate-specific antigen (tPSA) and free prostate-specific antigen (fPSA) were analyzed using linear models, adjusting for age. Log transformations were conducted for variables that were not normally distributed (fPSA, tPSA, TPV and IPSS). SNP association with aggressive *vs.* nonaggressive phenotypes was also evaluated using logistic regression with adjustment for age. Stratified analyses were conducted according to age, TPV, and IPSS scores. Subjects were separated at a median value for age and TPV and separated for IPSS at the BPH severity threshold (IPSS ≥ 19). All analyses were conducted using PLINK software [18]. *p*-values were two-tailed. An alpha of 0.05 was used to claim statistical significance.

## 4. Conclusions

In conclusion, we identified the significant association of rs103294 on *LILRA3* gene and BPH risk. Our findings demonstrate the significance of inflammation in the progression of BPH and the common genetic influences between PCa and BPH. Additional studies need to be conducted in the Chinese population to further evaluate our findings.

## Figures and Tables

**Table 1:** Clinical and demographic characteristics of all subjects.

	Cases		Controls

	N = 426		N = 1008
	Aggressive (N = 184)	Non-Aggressive (N = 242)	
Age			
Mean (SD)	73.84 (7.97)	70.45 (7.44)	61.24 (8.96)

PSA			
tPSA (ng/mL)			
<4 (%)	107 (58.2)	139 (57.4)	N/A
≥4 (%)	77 (41.8)	103 (42.6)	N/A

fPSA 1 (%)			
<25% fPSA	125 (67.9)	100 (41.3)	N/A
≥25% fPSA	58 (31.5)	141 (58.3)	N/A

TPV 2			
1st Quartile	50	62	N/A
2nd Quartile	70	74	N/A
3rd Percentile	90	90	N/A

IPSS 3			
Mean (SD)	18 (6.3)	14 (6.2)	N/A

1 No fPSA phenotype for 1 aggressive BPH case;

2 TPV = Total Prostate Volume;

3 IPSS = International Prostate Symptom Score.

**Table 2:** Association results for rs103294 on *LILRA3* and benign prostatic hyperplasia (BPH)/Aggressiveness risk.

					MAF		OR (95% CI) 3				

SNP	Chr	BP 1	Alleles 2	Risk allele	Cases	Controls	Homozygous non-risk genotypes	Heterozygous risk genotype	Homozygous risk genotype	Additive model	*p*-value 4
rs103294	19	54,797,848	C/T	C	0.289	0.242	1.00	1.37(1.04–1.81)	1.73(1.01–2.96)	1.34(1.09– 1.66)	0.0067
					Aggressive BPH	Non-Aggressive BPH					
rs103294	19	54,797,848	C/T	C	0.282	0.293	1.00	0.91(0.60–1.38)	1.46(0.66–3.25)	0.94 (0.69–1.29)	0.71

1 BP: Base Pair; based on NCBI Build 36;

2 Alleles are indicated by minor/major alleles;

3 OR and P are calculated based on logistic regression adjusting for age;

4 *p*-values are based on additive models.

**Table 3:** Association results for rs103294 on *LILRA3* and clinicopathological traits.

Traits	Allele 1	β (SE) 2	Quantitative means 3			*p*-value 4

			aa	aA	AA	
IPSS	C/T	−0.01(0.03)	14.15	14.51	14.61	0.70
TPV (mL)	C/T	−0.02(0.03)	66.09	71.45	71.52	0.41
tPSA (ng/mL))	C/T	−0.06(0.08)	3.71	3.73	3.38	0.16
fPSA (ng/mL)	C/T	0.07(0.08)	0.79	0.75	0.69	0.39

1 Alleles are indicated by minor/major alleles;

2 Beta and standard error results based on log-transformed data for IPSS, tPSA, fPSA and TPV;

3 aa indicates homozygous carriers of minor alleles, aA indicates heterozygous carriers, and AA indicates homozygous carriers of major alleles. Means were back-transformed;

4 *p*-values calculated using linear regression, assuming additive model, adjusting for age.

**Table 4:** Stratified analysis for association with BPH risk based on age, total prostate volume (TPV) and International Prostate Symptom Score (IPSS).

Phenotype	BPH cases (%)	rs103294	

		OR 1 (95% CI)	*p*-value 1
Age			
<72 yrs	201(47.2)	1.51(1.06–2.16)	0.022
≥72 yrs	225(52.8)	1.27(0.99–1.62)	0.060

TPV			
<72 mL	207(48.6)	1.35(1.03–1.77)	0.028
≥72 mL	219(51.4)	1.37(1.06–1.79)	0.018

IPSS			
<19	300(70.4)	1.44(1.02–2.04)	0.038
≥19	126(29.6)	1.35(1.07–1.70)	0.011

1 OR and *p* are calculated based on logistic regression, adjusting for age.
